# Supplementary material for: Alzheimer’s Disease Risk Allele APOE4 Interacts with Arsenic Exposure to Drive Microglial Dysfunction
Source: bioRxiv. 2026 May 12:2026.05.09.723490. Preprint. [Version 1] doi: 10.64898/2026.05.09.723490 (PMC13192950; doi:10.64898/2026.05.09.723490)
Supplement: Supplement 1 [file media-1.pdf]

Supplemental Figure Legends:

Supplementary Figure 1: (A) Sanger sequencing of APOE3+/+ control and APOE4+/+ monoclones aligned to the endogenous APOE4 locus with representative chromatogram shown. (B) Ven diagram depicting shared and novel mismatches to hg38 from whole genome sequencing of two APOE4+/+ and the parental APOE3+/+ cell line. (C) Graph depicting the type and proportion of mutations detected from whole genome sequencing. (D) Heatmap of gene expression changes between APOE3+/+ and APOE4+/+ iTFMs on differentiation day 8. (E) Volcano plot of differentially expressed genes in APOE4+/+ compared to APOE3+/+.

Supplementary Figure 2: Volcano plots of 72 hr arsenite treated APOE3+/+ iTFM compared to vehicle treated APOE3+/+ iTFM.

Supplementary Figure 3: Volcano plots of 72 hr arsenite treated APOE4+/+ iTFM compared to vehicle treated APOE4+/+ iTFM.

Supplementary Figure 4: Volcano plots of 72 hr vehicle and arsenite treated APOE4+/+ iTFM compared to the respective APOE3+/+ control iTFM.

Supplementary Figure 5: (A-F) Ingenuity pathway analysis of APOE4+/+ differentially expressed genes (absolute fold-change ≥2, padj < 0.05) when treated with increasing

concentrations of arsenite for 72 hrs (top to bottom). Shown are top enriched pathways (left), top predicted upstream regulators (middle), and top predicted cellular functions (right).

Supplementary Figure 6: PCA of weighted gene co-expression network analysis for APOE3+/+ (blue) and APOE4+/+ (yellow). Each dot represents an independent biological sample responding to increasing concentration of arsenite (light to dark).

Supplementary Figure 7: Mean fluorescent intensity of (C-D) APOE3+/+ (purple) and APOE4+/+ (teal) iTFM. Statistical significance assessed by two-way ANOVA followed by Bonferroni post hoc testing. *, **, and *** correspond to p values <0.05, 0.005, 0.0001 respectively.

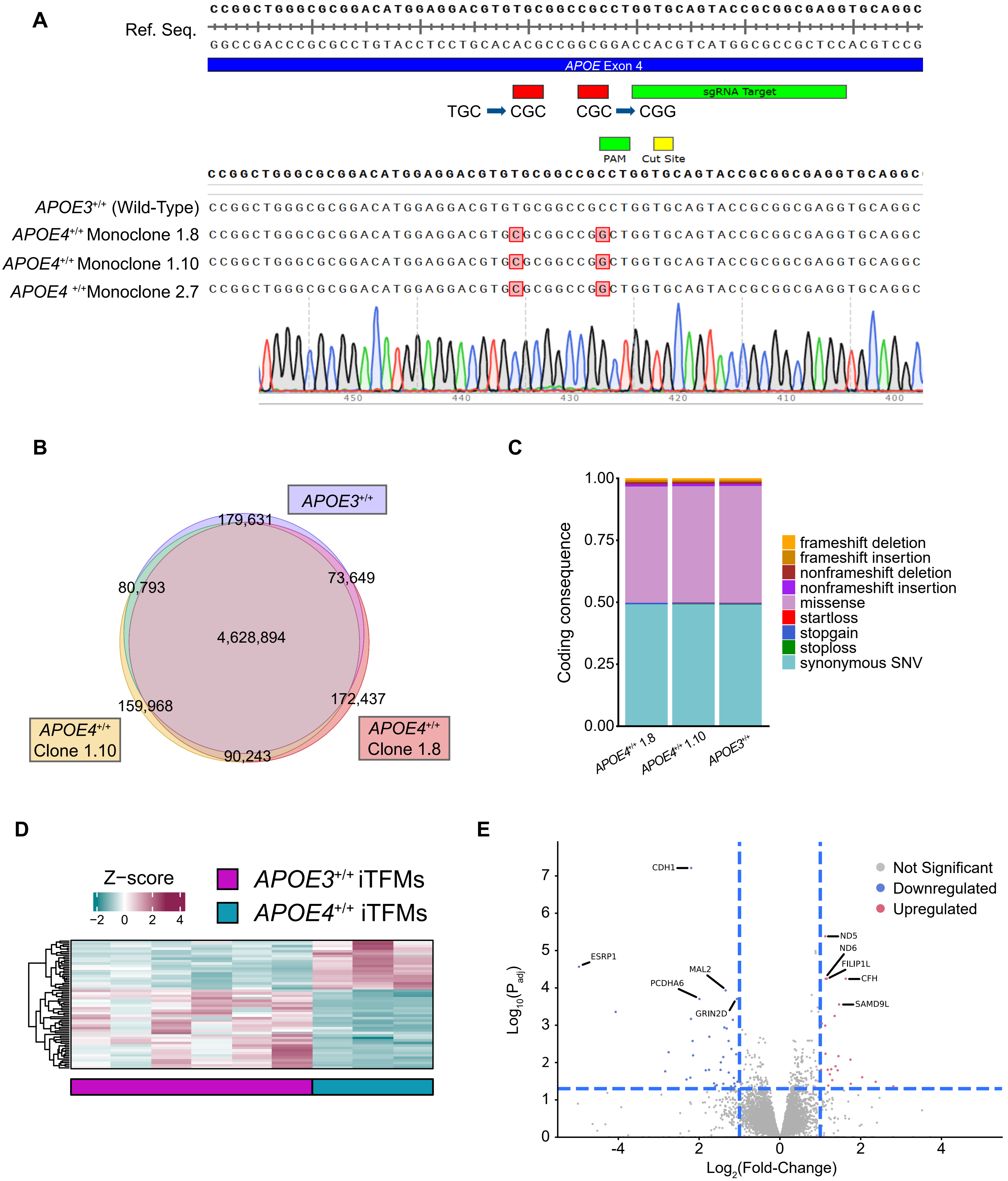
A
Ref. Seq.
CCGGCTGGGCGCGGACATGGAGGACGTGTGCGGCCGCCTGGTGCAGTACCGCGGCGAGGTGCAGGC
GGCCGACCCGCGCCTGTACCTCCTGCACACGCCGGCGGACCACGTCATGGCGCCGCTCCACGTCCG
APOE Exon 4
sgRNA Target
TGC ➡ CGC
CGC ➡ CGG
PAM
Cut Site
CCGGCTGGGCGCGGACATGGAGGACGTGTGCGGCCGCCTGGTGCAGTACCGCGGCGAGGTGCAGGC
APOE3+/+ (Wild-Type)
CCGGCTGGGCGCGGACATGGAGGACGTGTGCGGCCGCCTGGTGCAGTACCGCGGCGAGGTGCAGGC
APOE4+/+ Monoclone 1.8
CCGGCTGGGCGCGGACATGGAGGACGTGCGCGGCCGGCTGGTGCAGTACCGCGGCGAGGTGCAGGC
APOE4+/+ Monoclone 1.10
CCGGCTGGGCGCGGACATGGAGGACGTGCGCGGCCGGCTGGTGCAGTACCGCGGCGAGGTGCAGGC
APOE4 +/+Monoclone 2.7
CCGGCTGGGCGCGGACATGGAGGACGTGCGCGGCCGGCTGGTGCAGTACCGCGGCGAGGTGCAGGC
450
440
430
420
410
400
B
APOE3+/+
179,631
80,793
73,649
4,628,894
159,968
172,437
90,243
APOE4+/+ Clone 1.10
APOE4+/+ Clone 1.8
C
Coding consequence
1.00
0.75
0.50
0.25
0.00
APOE4+/+ 1.8
APOE4+/+ 1.10
APOE3+/+
frameshift deletion
frameshift insertion
nonframeshift deletion
nonframeshift insertion
missense
startloss
stopgain
stoploss
synonymous SNV
D
Z−score
−2 0 2 4
APOE3+/+ iTFMs
APOE4+/+ iTFMs
E
Log10(Padj)
Log2(Fold-Change)
-4
2
0
2
4
Not Significant
Downregulated
Upregulated
CDH1
ESRP1
MAL2
PCDHA6
GRIN2D
ND5
ND6
FILIP1L
CFH
SAMD9L

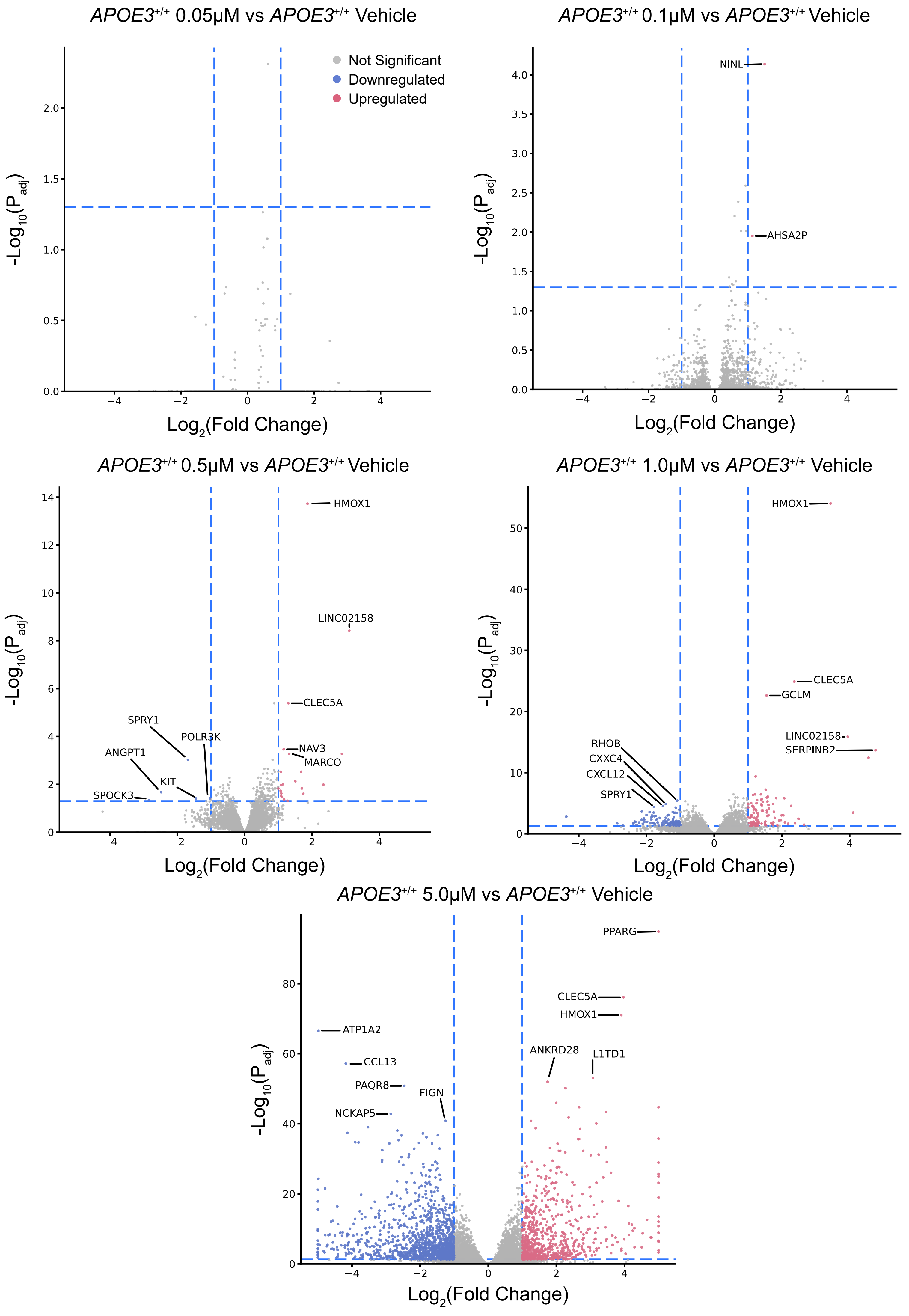
APOE3+/+ 0.05μM vs APOE3+/+ Vehicle
Not Significant
Downregulated
Upregulated
-Log10(Padj)
Log2(Fold Change)
APOE3+/+ 0.1μM vs APOE3+/+ Vehicle
NINL
AHSA2P
-Log10(Padj)
Log2(Fold Change)
APOE3+/+ 0.5μM vs APOE3+/+ Vehicle
HMOX1
LINC02158
CLEC5A
SPRY1
POLR3K
ANGPT1
NAV3
MARCO
KIT
SPOCK3
-Log10(Padj)
Log2(Fold Change)
APOE3+/+ 1.0μM vs APOE3+/+ Vehicle
HMOX1
CLEC5A
GCLM
LINC02158
SERPINB2
RHOB
CXXC4
CXCL12
SPRY1
-Log10(Padj)
Log2(Fold Change)
APOE3+/+ 5.0μM vs APOE3+/+ Vehicle
PPARG
CLEC5A
HMOX1
ATP1A2
ANKRD28
L1TD1
CCL13
PAQR8
FIGN
NCKAP5
-Log10(Padj)
Log2(Fold Change)

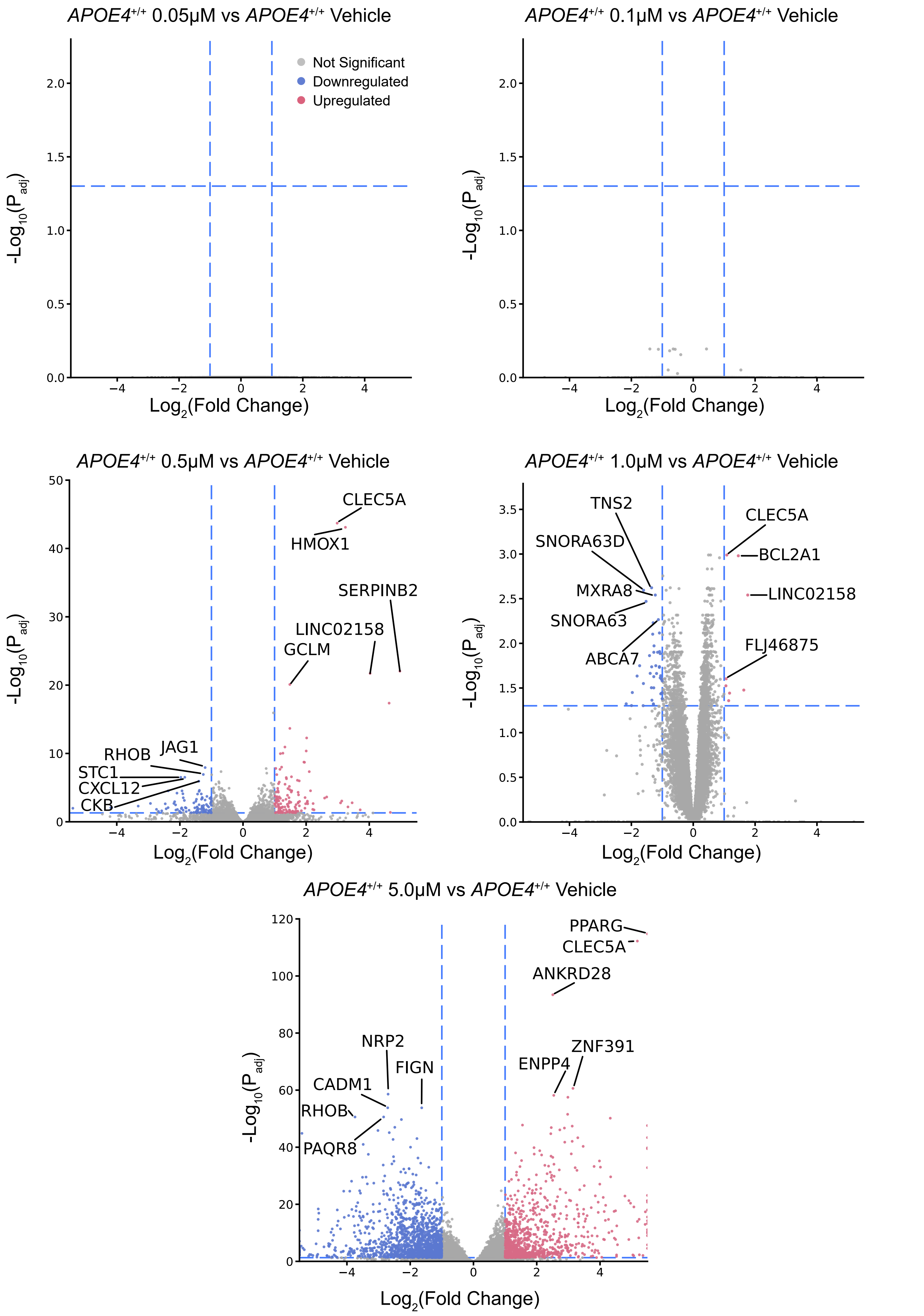

APOE4+/+ 0.05μM vs APOE4+/+ Vehicle
Not Significant
Downregulated
Upregulated
-Log10(Padj)
Log2(Fold Change)
APOE4+/+ 0.1μM vs APOE4+/+ Vehicle
-Log10(Padj)
Log2(Fold Change)
APOE4+/+ 0.5μM vs APOE4+/+ Vehicle
CLEC5A
HMOX1
SERPINB2
LINC02158
GCLM
JAG1
RHOB
STC1
CXCL12
CKB
-Log10(Padj)
Log2(Fold Change)
APOE4+/+ 1.0μM vs APOE4+/+ Vehicle
TNS2
SNORA63D
MXRA8
SNORA63
ABCA7
CLEC5A
BCL2A1
LINC02158
FLJ46875
-Log10(Padj)
Log2(Fold Change)
APOE4+/+ 5.0μM vs APOE4+/+ Vehicle
PPARG
CLEC5A
ANKRD28
NRP2
FIGN
CADM1
RHOB
PAQR8
ENPP4
ZNF391
-Log10(Padj)
Log2(Fold Change)

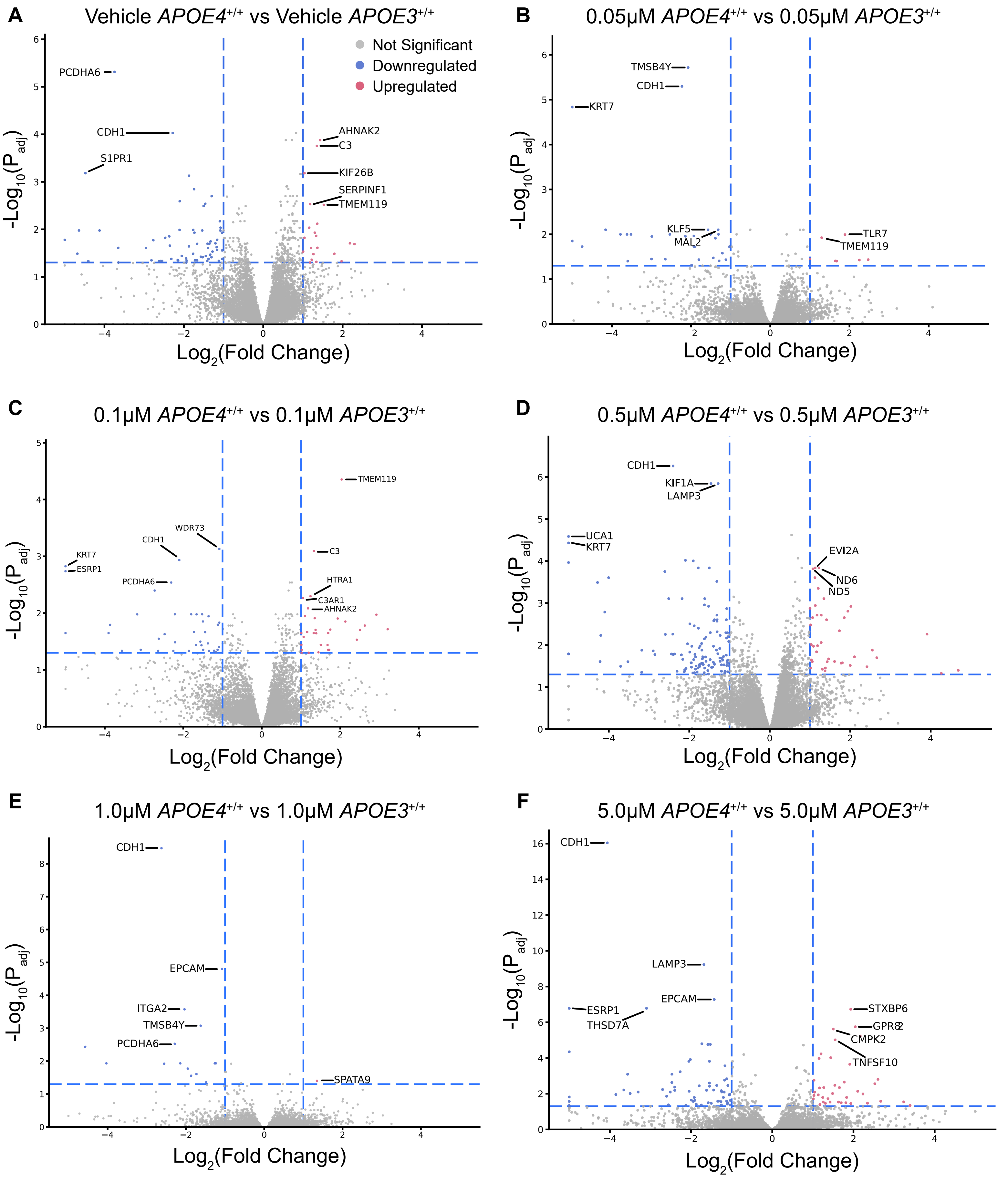

A
Vehicle APOE4+/+ vs Vehicle APOE3+/+
Not Significant
Downregulated
Upregulated
PCDHA6
CDH1
S1PR1
AHNAK2
C3
KIF26B
SERPINF1
TMEM119
-Log10(Padj)
Log2(Fold Change)
B
0.05µM APOE4+/+ vs 0.05µM APOE3+/+
TMSB4Y
CDH1
KRT7
KLF5
MAL2
TLR7
TMEM119
C
0.1µM APOE4+/+ vs 0.1µM APOE3+/+
TMEM119
WDR73
CDH1
KRT7
ESRP1
PCDHA6
C3
HTRA1
C3AR1
AHNAK2
D
0.5µM APOE4+/+ vs 0.5µM APOE3+/+
CDH1
KIF1A
LAMP3
UCA1
KRT7
EVI2A
ND6
ND5
E
1.0µM APOE4+/+ vs 1.0µM APOE3+/+
CDH1
EPCAM
ITGA2
TMSB4Y
PCDHA6
SPATA9
F
5.0µM APOE4+/+ vs 5.0µM APOE3+/+
CDH1
LAMP3
EPCAM
ESRP1
THSD7A
STXBP6
GPR82
CMPK2
TNFSF10

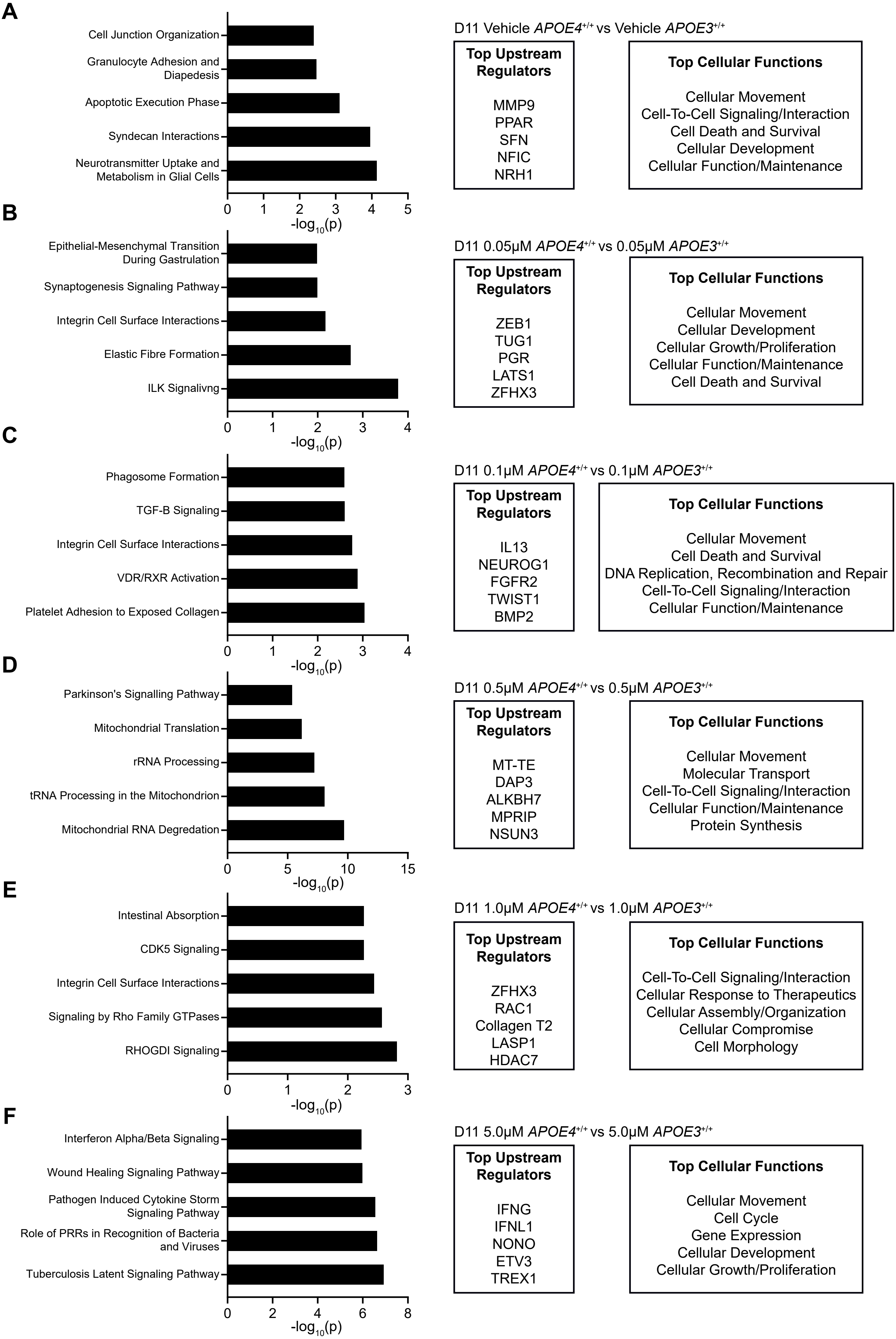
A
Cell Junction Organization
Granulocyte Adhesion and Diapedesis
Apoptotic Execution Phase
Syndecan Interactions
Neurotransmitter Uptake and Metabolism in Glial Cells
0 1 2 3 4 5
-log10(p)
D11 Vehicle APOE4+/+ vs Vehicle APOE3+/+
Top Upstream Regulators
MMP9
PPAR
SFN
NFIC
NRH1
Top Cellular Functions
Cellular Movement
Cell-To-Cell Signaling/Interaction
Cell Death and Survival
Cellular Development
Cellular Function/Maintenance
B
Epithelial-Mesenchymal Transition During Gastrulation
Synaptogenesis Signaling Pathway
Integrin Cell Surface Interactions
Elastic Fibre Formation
ILK Signalivng
0 1 2 3 4
-log10(p)
D11 0.05μM APOE4+/+ vs 0.05μM APOE3+/+
Top Upstream Regulators
ZEB1
TUG1
PGR
LATS1
ZFHX3
Top Cellular Functions
Cellular Movement
Cellular Development
Cellular Growth/Proliferation
Cellular Function/Maintenance
Cell Death and Survival
C
Phagosome Formation
TGF-B Signaling
Integrin Cell Surface Interactions
VDR/RXR Activation
Platelet Adhesion to Exposed Collagen
0 1 2 3 4
-log10(p)
D11 0.1μM APOE4+/+ vs 0.1μM APOE3+/+
Top Upstream Regulators
IL13
NEUROG1
FGFR2
TWIST1
BMP2
Top Cellular Functions
Cellular Movement
Cell Death and Survival
DNA Replication, Recombination and Repair
Cell-To-Cell Signaling/Interaction
Cellular Function/Maintenance
D
Parkinson's Signalling Pathway
Mitochondrial Translation
rRNA Processing
tRNA Processing in the Mitochondrion
Mitochondrial RNA Degredation
0 5 10 15
-log10(p)
D11 0.5μM APOE4+/+ vs 0.5μM APOE3+/+
Top Upstream Regulators
MT-TE
DAP3
ALKBH7
MPRIP
NSUN3
Top Cellular Functions
Cellular Movement
Molecular Transport
Cell-To-Cell Signaling/Interaction
Cellular Function/Maintenance
Protein Synthesis
E
Intestinal Absorption
CDK5 Signaling
Integrin Cell Surface Interactions
Signaling by Rho Family GTPases
RHOGDI Signaling
0 1 2 3
-log10(p)
D11 1.0μM APOE4+/+ vs 1.0μM APOE3+/+
Top Upstream Regulators
ZFHX3
RAC1
Collagen T2
LASP1
HDAC7
Top Cellular Functions
Cell-To-Cell Signaling/Interaction
Cellular Response to Therapeutics
Cellular Assembly/Organization
Cellular Compromise
Cell Morphology
F
Interferon Alpha/Beta Signaling
Wound Healing Signaling Pathway
Pathogen Induced Cytokine Storm Signaling Pathway
Role of PRRs in Recognition of Bacteria and Viruses
Tuberculosis Latent Signaling Pathway
0 2 4 6 8
-log10(p)
D11 5.0μM APOE4+/+ vs 5.0μM APOE3+/+
Top Upstream Regulators
IFNG
IFNL1
NONO
ETV3
TREX1
Top Cellular Functions
Cellular Movement
Cell Cycle
Gene Expression
Cellular Development
Cellular Growth/Proliferation

A

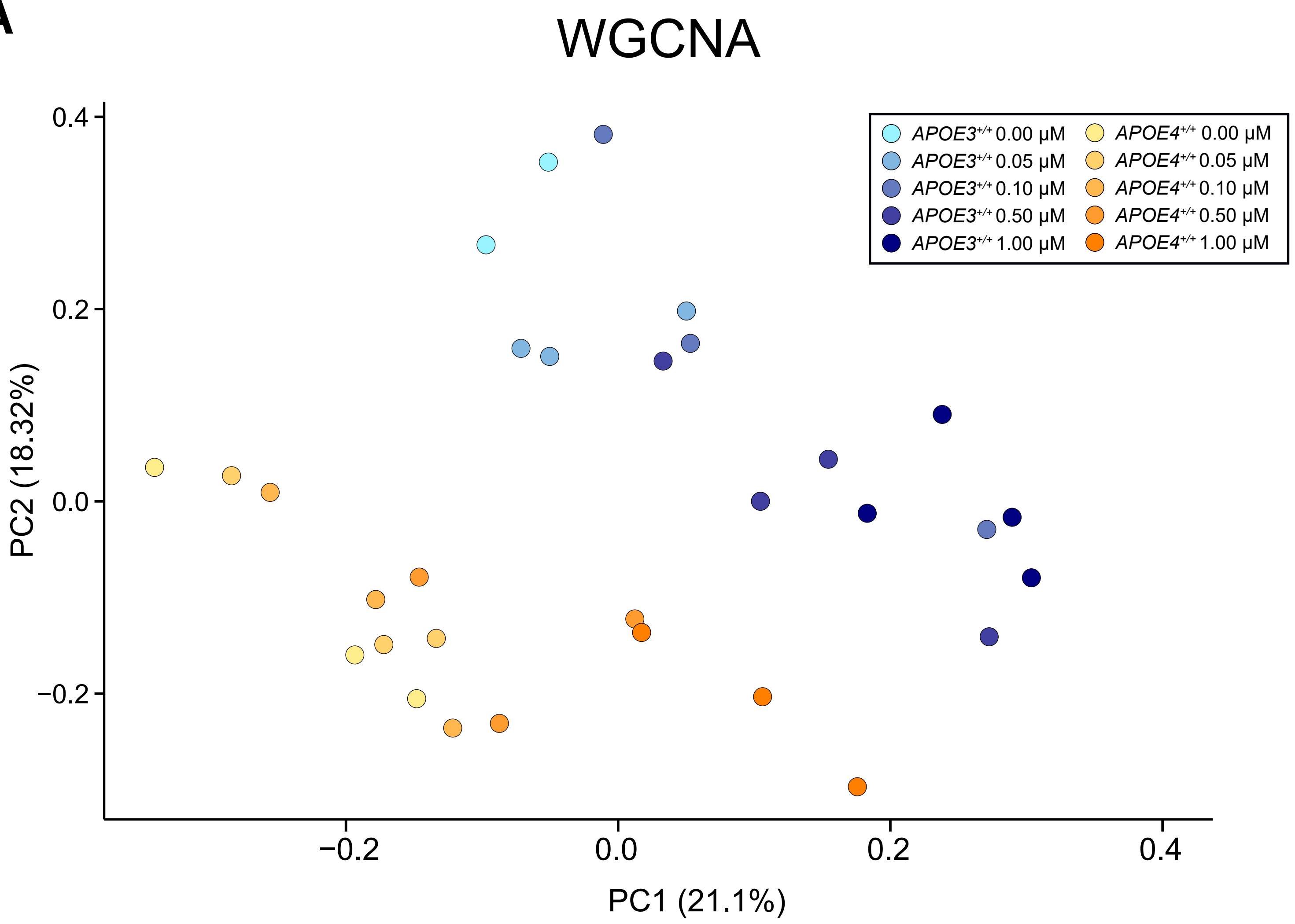

WGCNA
PC2 (18.32%)
PC1 (21.1%)
0.4
0.2
0.0
−0.2
−0.2
0.0
0.2
0.4
APOE3+/+ 0.00 µM
APOE3+/+ 0.05 µM
APOE3+/+ 0.10 µM
APOE3+/+ 0.50 µM
APOE3+/+ 1.00 µM
APOE4+/+ 0.00 µM
APOE4+/+ 0.05 µM
APOE4+/+ 0.10 µM
APOE4+/+ 0.50 µM
APOE4+/+ 1.00 µM

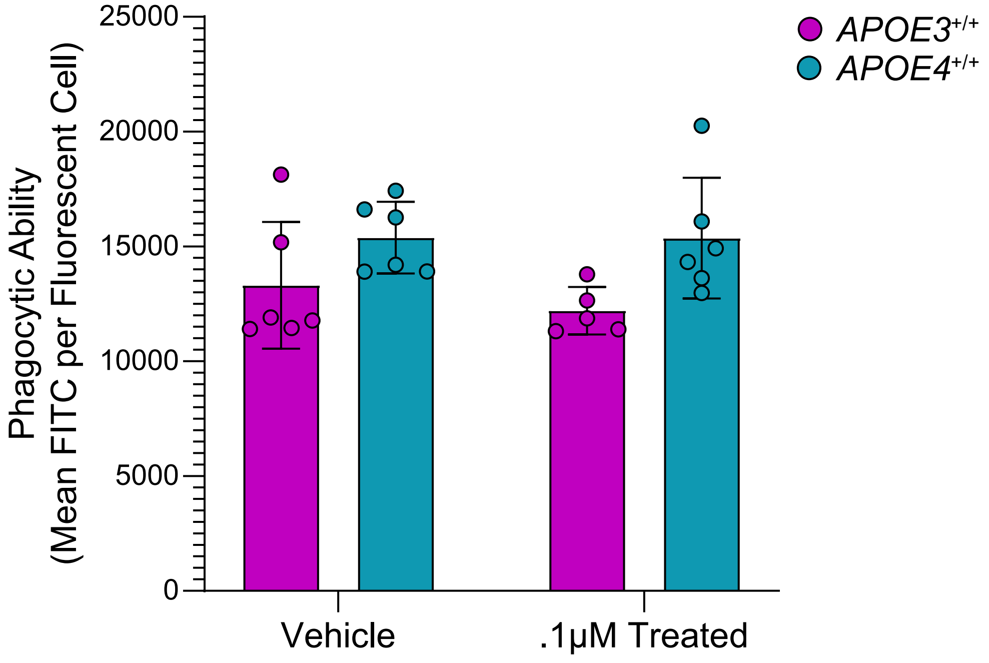

Phagocytic Ability
(Mean FITC per Fluorescent Cell)
25000
20000
15000
10000
5000
0
Vehicle
.1μM Treated
APOE3+/+
APOE4+/+
