# Supplementary material for: Alzheimer’s Disease Risk Allele APOE4 Interacts with Arsenic Exposure to Drive Microglial Dysfunction
Source: bioRxiv. 2026 May 12:2026.05.09.723490. Preprint. [Version 1] doi: 10.64898/2026.05.09.723490 (PMC13192950; doi:10.64898/2026.05.09.723490)
Supplement: Supplement 2 [file media-2.pdf]

Supplementary Figure 8: Heatmaps of assigned WGCNA modules showing relative expression changes across arsenite concentration. *APOE3*$^{+/+}$ in pink, *APOE4*$^{+/+}$ in blue.

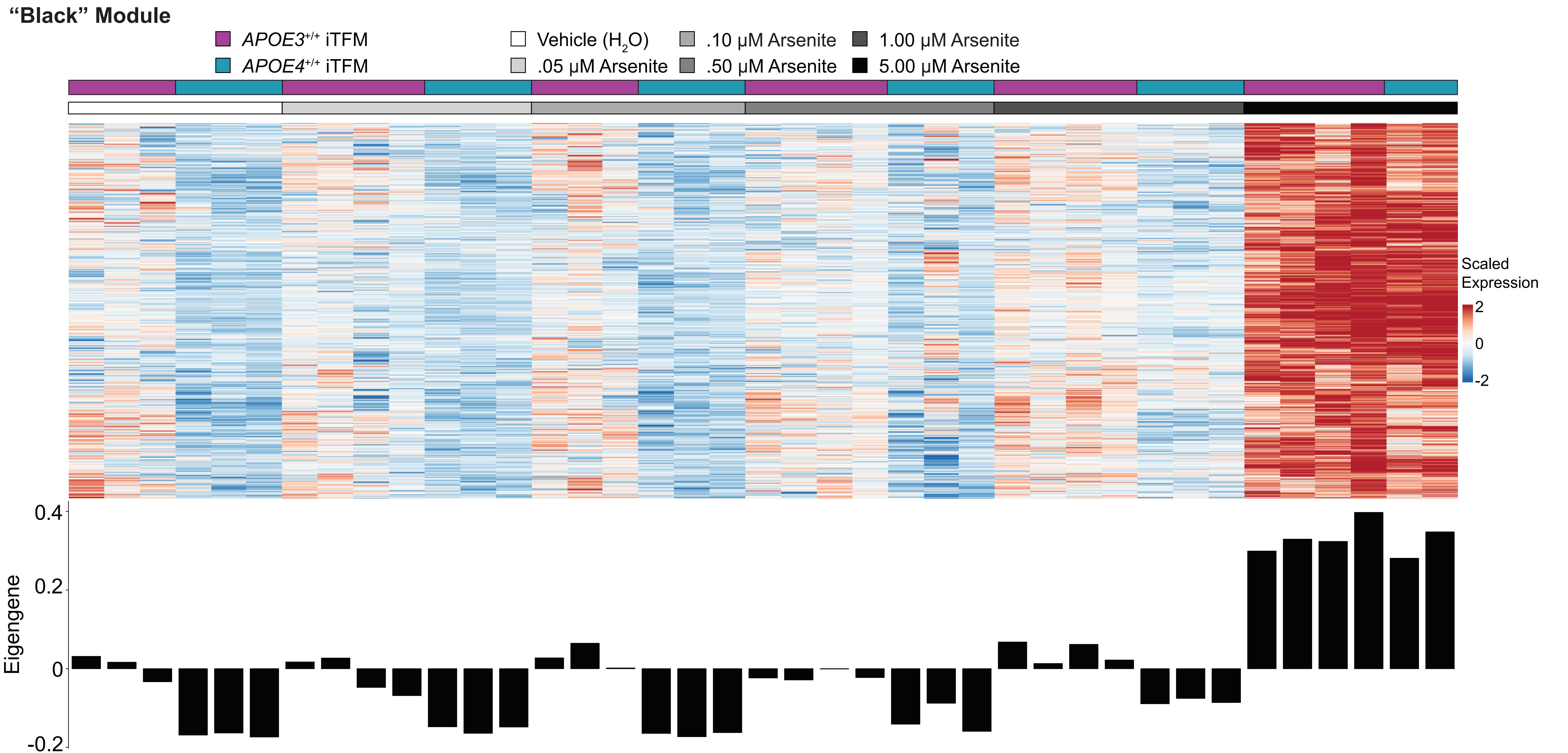

"Black" Module
APOE3+/+ iTFM
APOE4+/+ iTFM
Vehicle (H2O)
.05 μM Arsenite
.10 μM Arsenite
.50 μM Arsenite
1.00 μM Arsenite
5.00 μM Arsenite
Scaled Expression
2
0
-2
Eigengene
0.4
0.2
0
-0.2

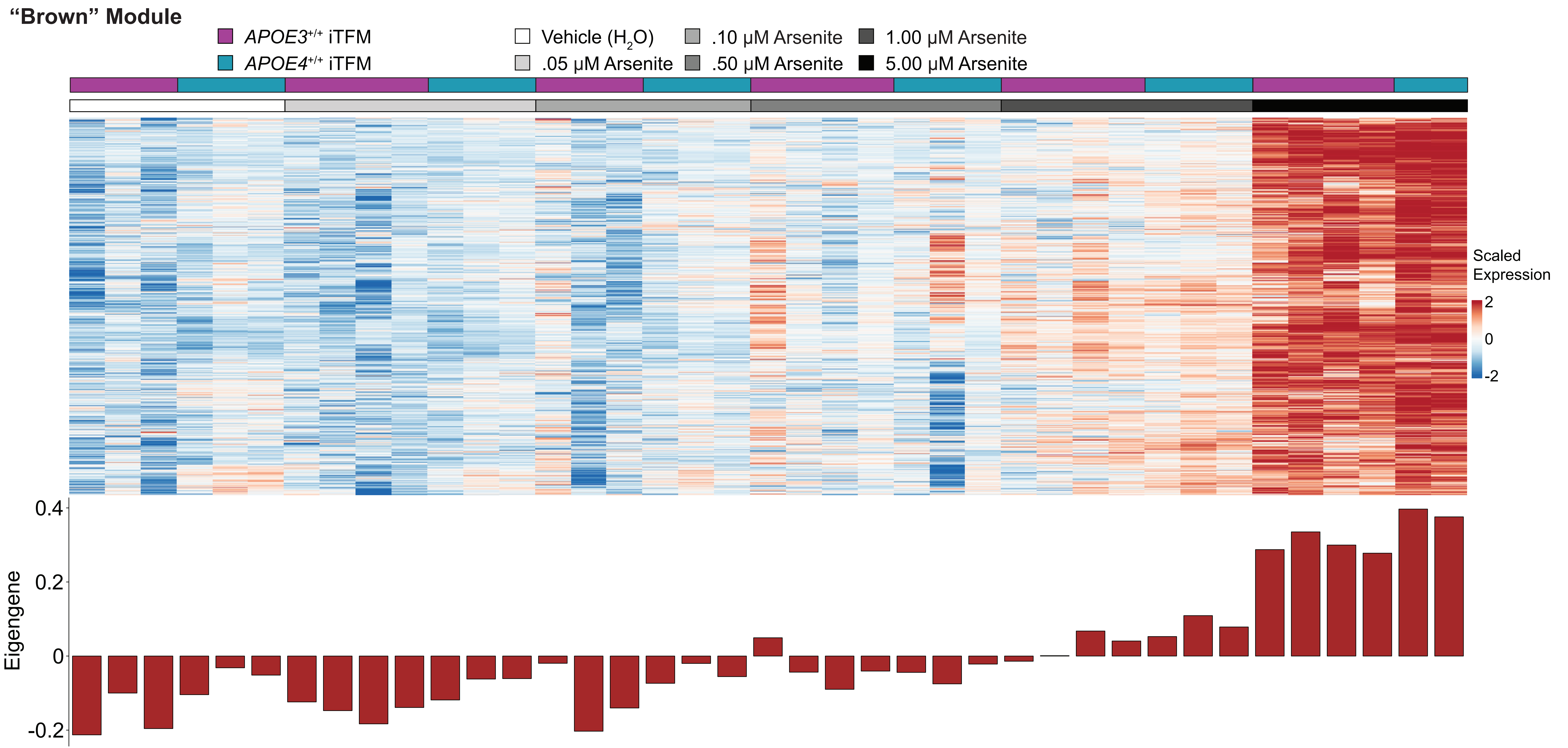

"Brown" Module
APOE3+/+ iTFM
APOE4+/+ iTFM
Vehicle (H2O)
.05 μM Arsenite
.10 μM Arsenite
.50 μM Arsenite
1.00 μM Arsenite
5.00 μM Arsenite
Scaled Expression
2
0
-2
Eigengene
0.4
0.2
0
-0.2

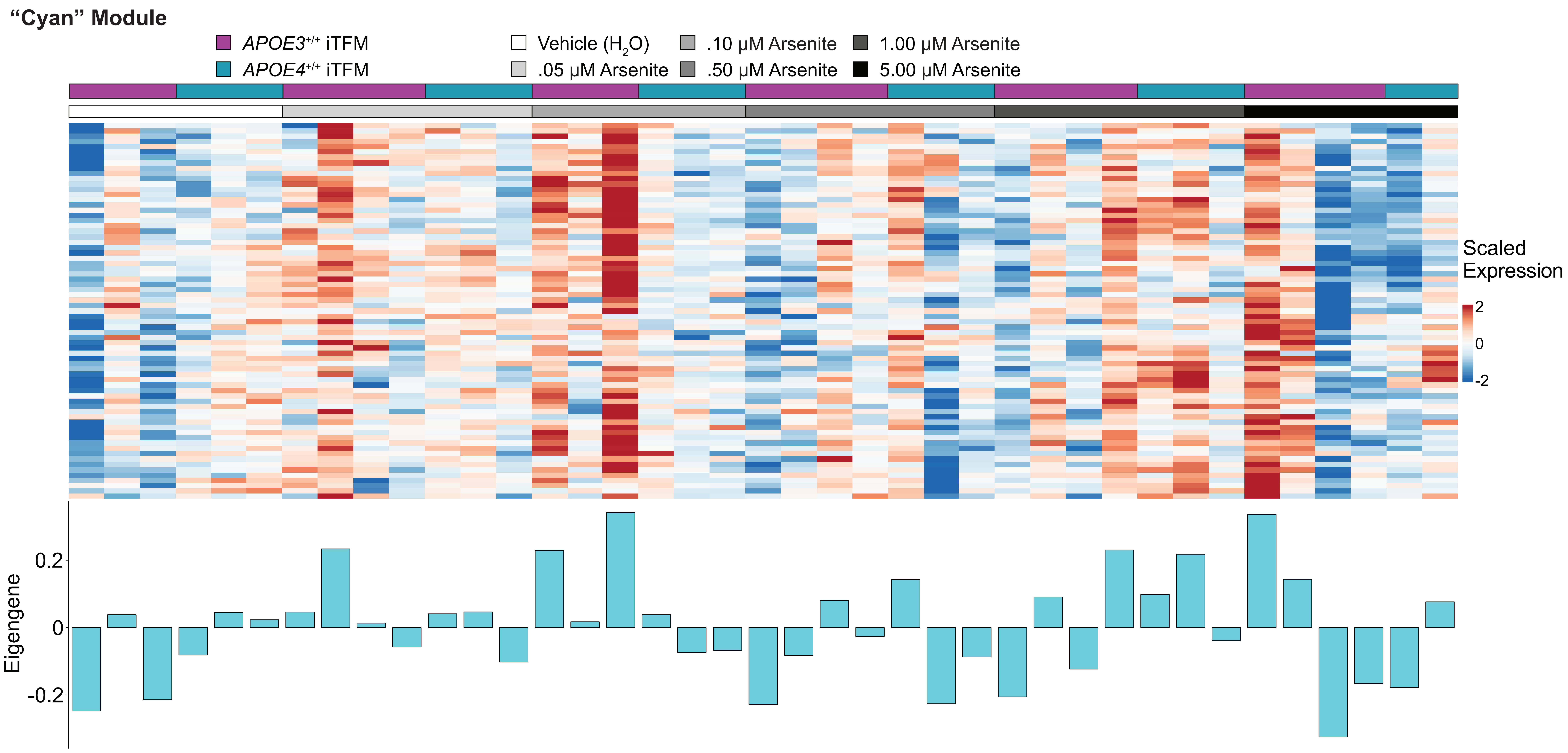
"Cyan" Module
APOE3+/+ iTFM
APOE4+/+ iTFM
Vehicle (H2O)
.05 μM Arsenite
.10 μM Arsenite
.50 μM Arsenite
1.00 μM Arsenite
5.00 μM Arsenite
Scaled Expression
2
0
-2
Eigengene
0.2
0
-0.2

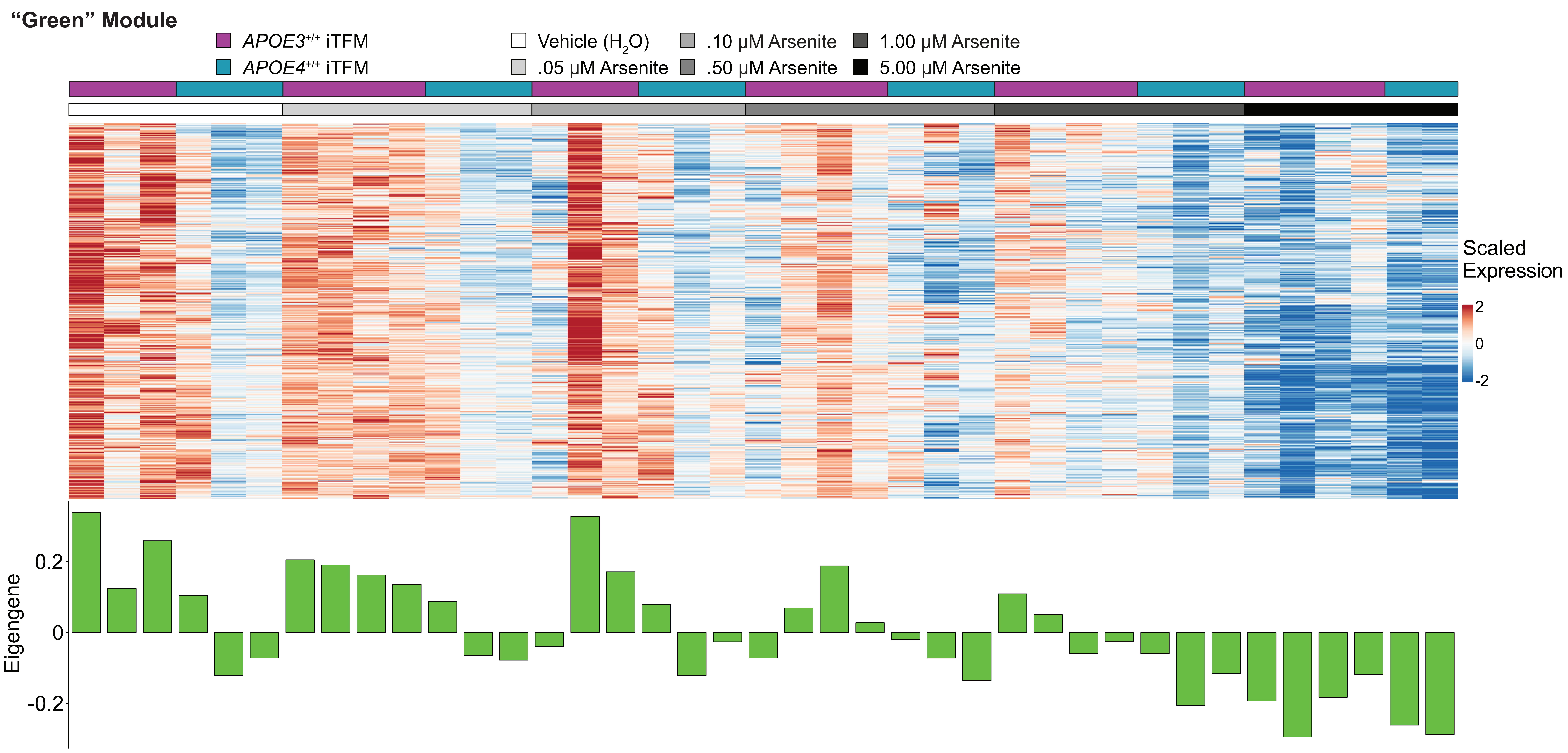
"Green" Module
APOE3+/+ iTFM
APOE4+/+ iTFM
Vehicle (H2O)
.05 μM Arsenite
.10 μM Arsenite
.50 μM Arsenite
1.00 μM Arsenite
5.00 μM Arsenite
Scaled Expression
2
0
-2
Eigengene
0.2
0
-0.2

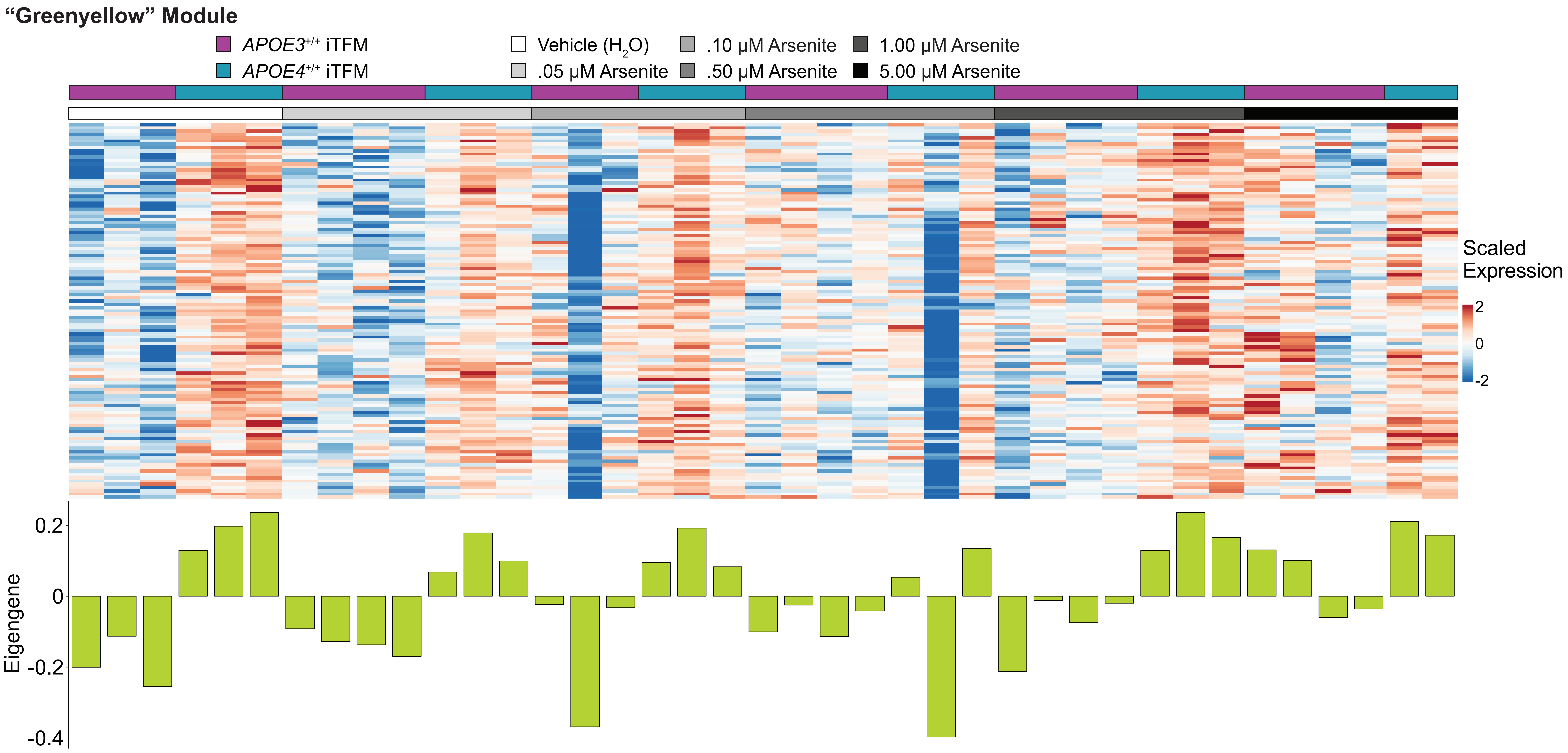
"Greenyellow" Module
APOE3+/+ iTFM
APOE4+/+ iTFM
Vehicle (H2O)
.05 μM Arsenite
.10 μM Arsenite
.50 μM Arsenite
1.00 μM Arsenite
5.00 μM Arsenite
Scaled Expression
2
0
-2
Eigengene
0.2
0
-0.2
-0.4

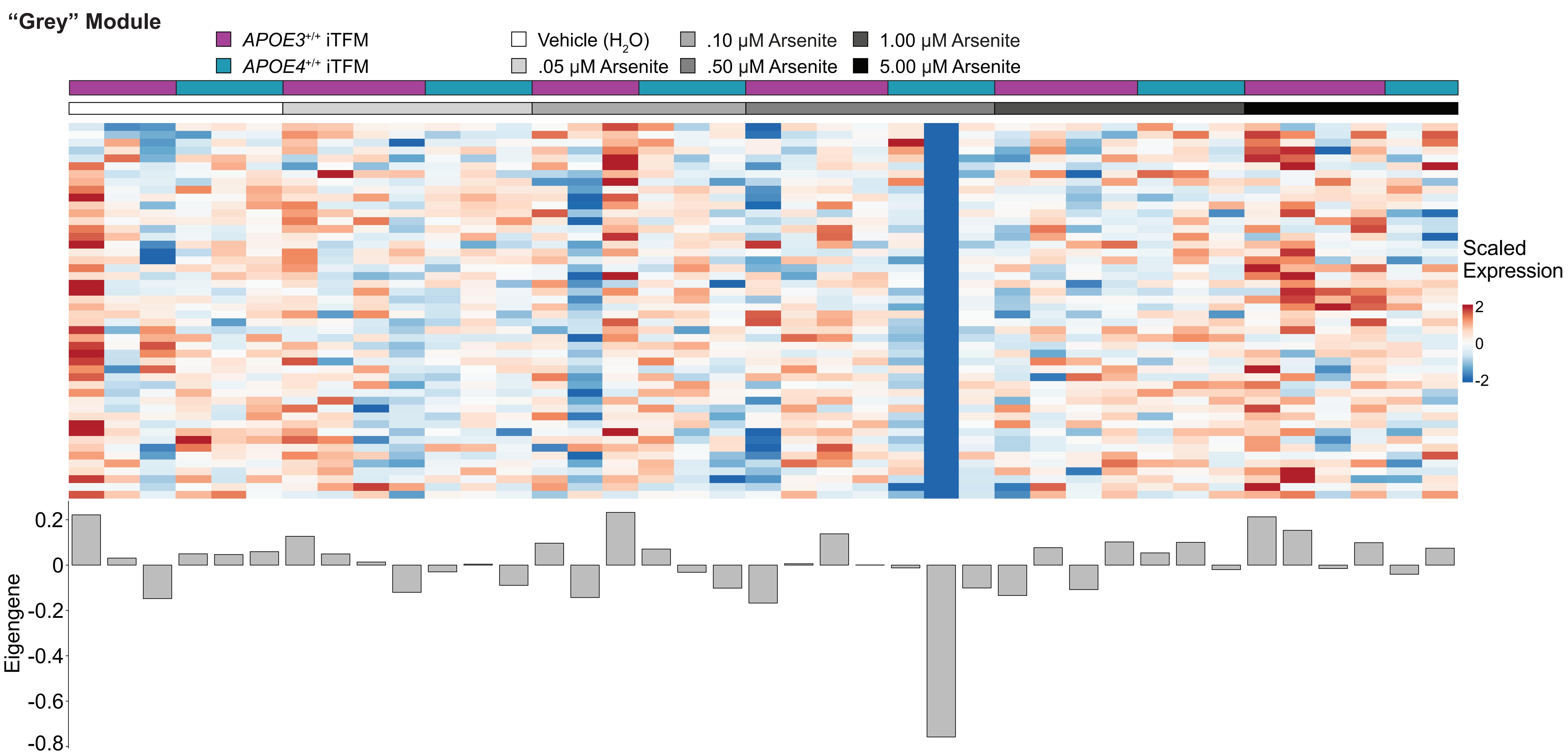
"Grey" Module
APOE3+/+ iTFM
APOE4+/+ iTFM
Vehicle (H2O)
.05 μM Arsenite
.10 μM Arsenite
.50 μM Arsenite
1.00 μM Arsenite
5.00 μM Arsenite
Scaled Expression
2
0
-2
Eigengene
0.2
0
-0.2
-0.4
-0.6
-0.8

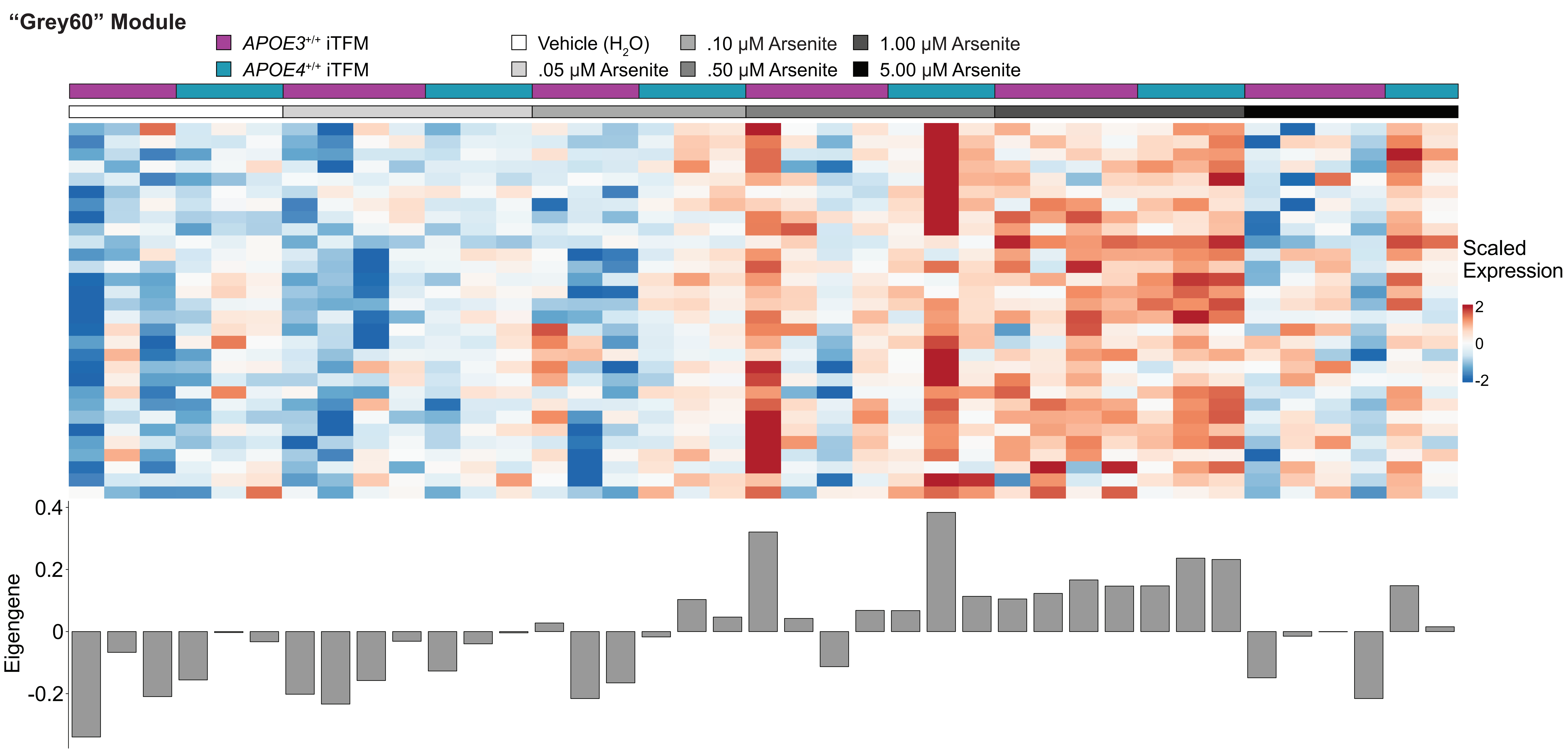

"Grey60" Module
APOE3+/+ iTFM
APOE4+/+ iTFM
Vehicle (H2O)
.05 μM Arsenite
.10 μM Arsenite
.50 μM Arsenite
1.00 μM Arsenite
5.00 μM Arsenite
Scaled Expression
2
0
-2
Eigengene
0.4
0.2
0
-0.2

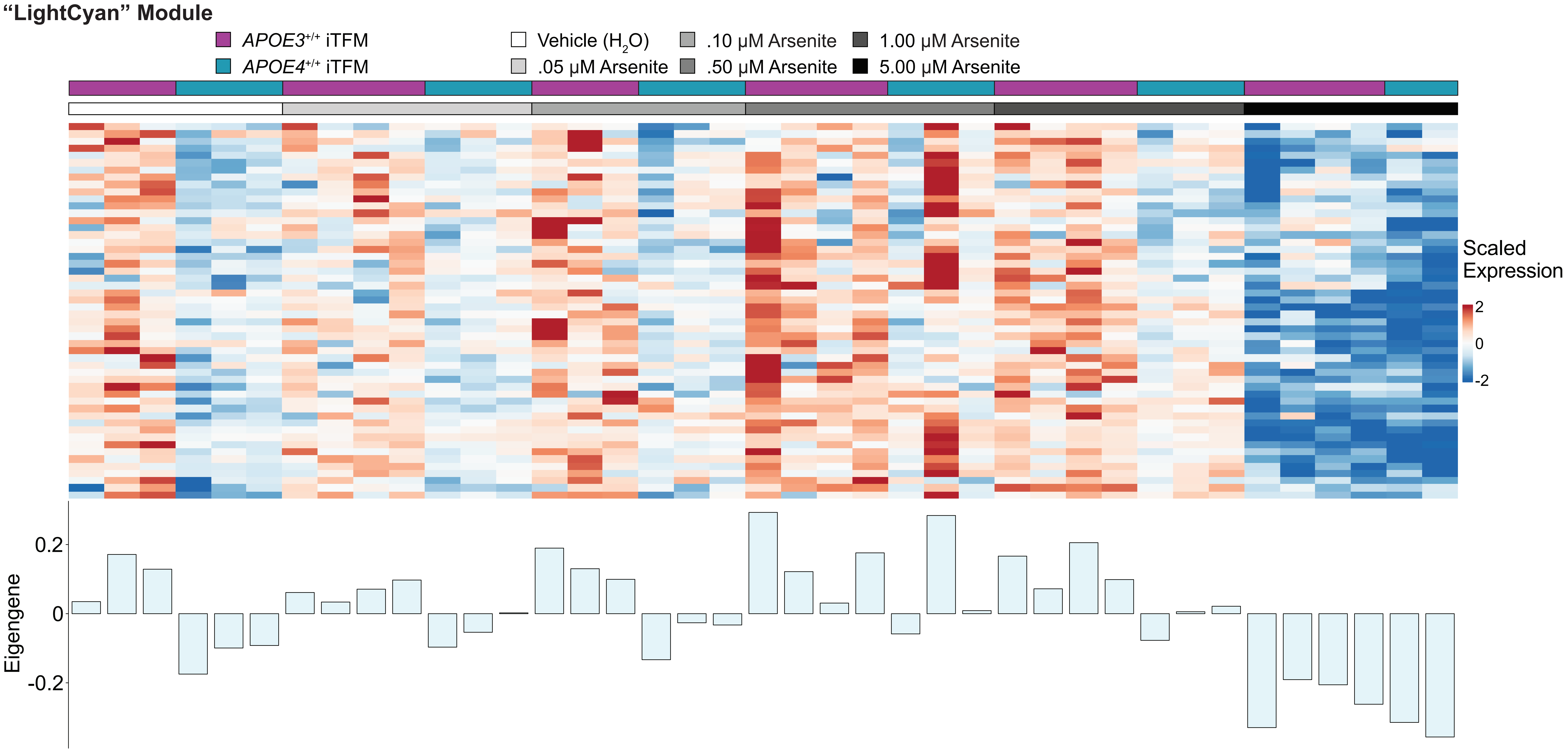
"LightCyan" Module
APOE3+/+ iTFM
APOE4+/+ iTFM
Vehicle (H2O)
.05 µM Arsenite
.10 µM Arsenite
.50 µM Arsenite
1.00 µM Arsenite
5.00 µM Arsenite
Scaled Expression
2
0
-2
Eigengene
0.2
0
-0.2

“Magenta” Module

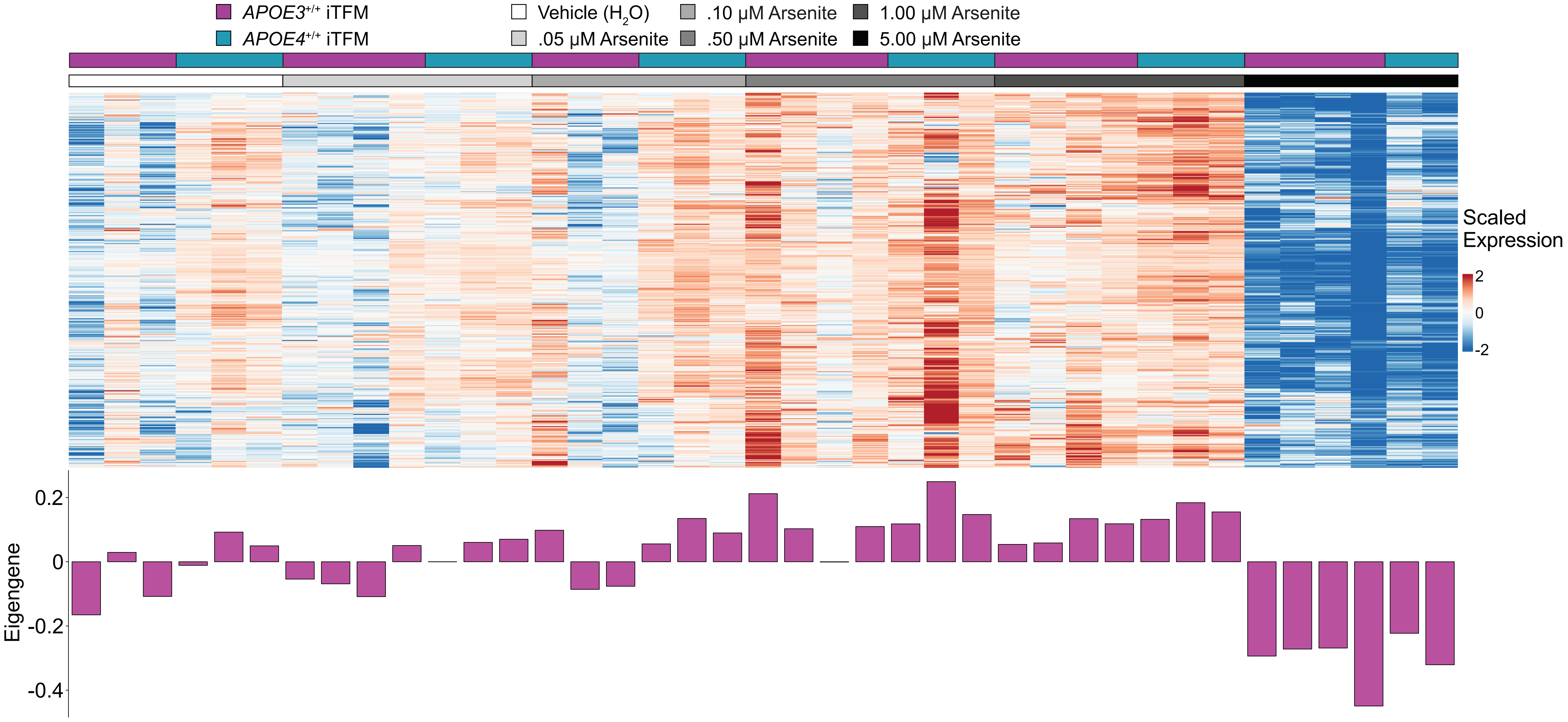

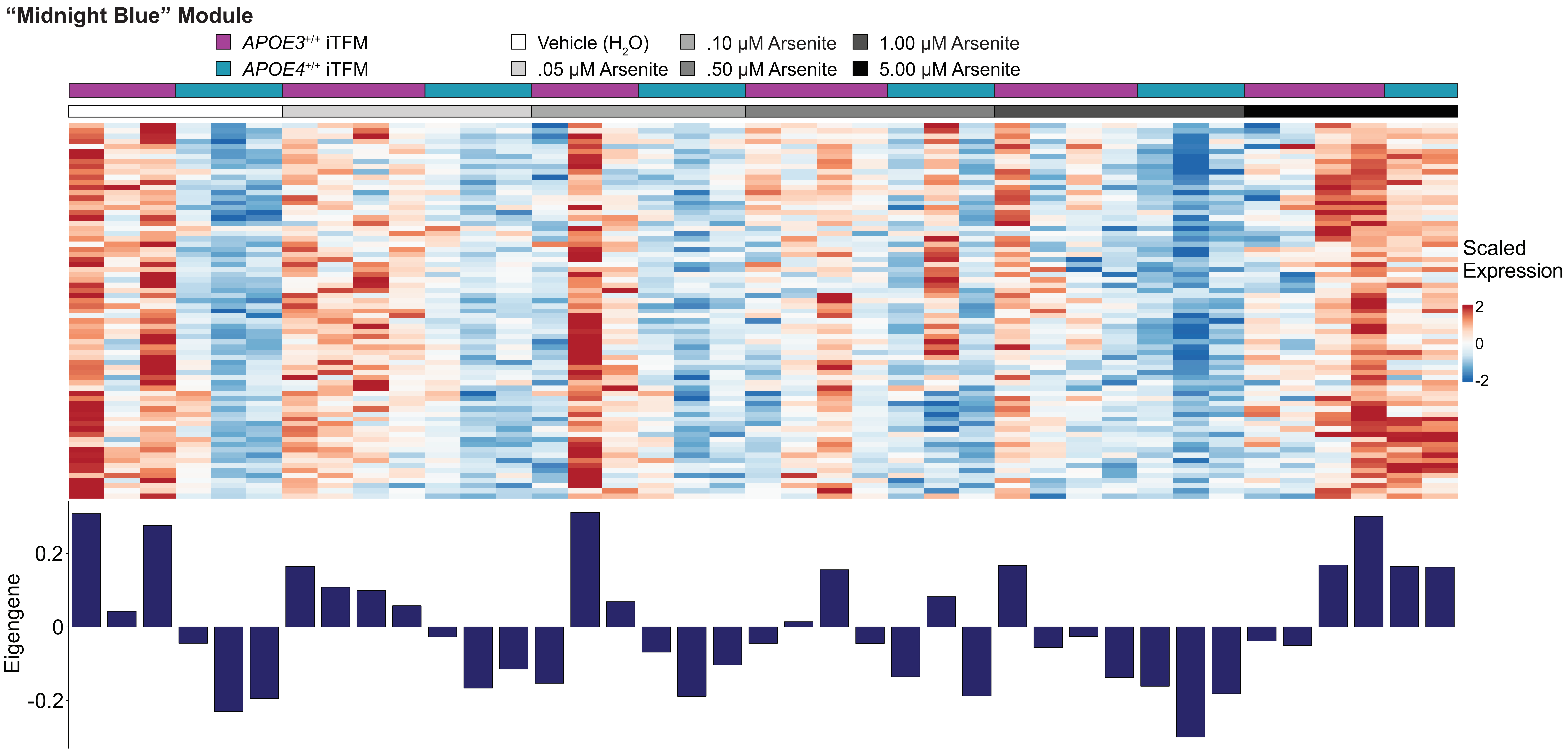
"Midnight Blue" Module
APOE3+/+ iTFM
APOE4+/+ iTFM
Vehicle (H2O)
.05 μM Arsenite
.10 μM Arsenite
.50 μM Arsenite
1.00 μM Arsenite
5.00 μM Arsenite
Scaled Expression
2
0
-2
Eigengene
0.2
0
-0.2

“Purple” Module

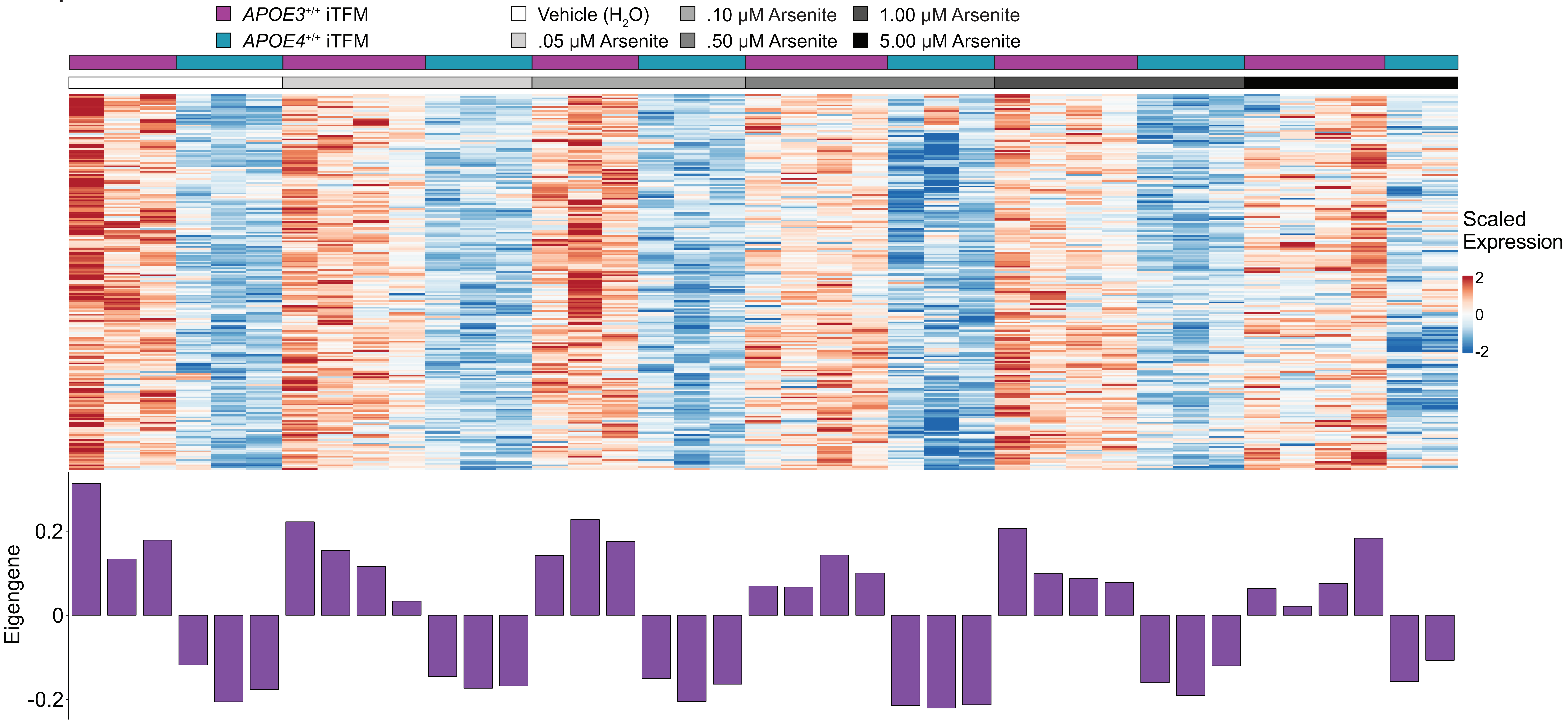

"Red" Module

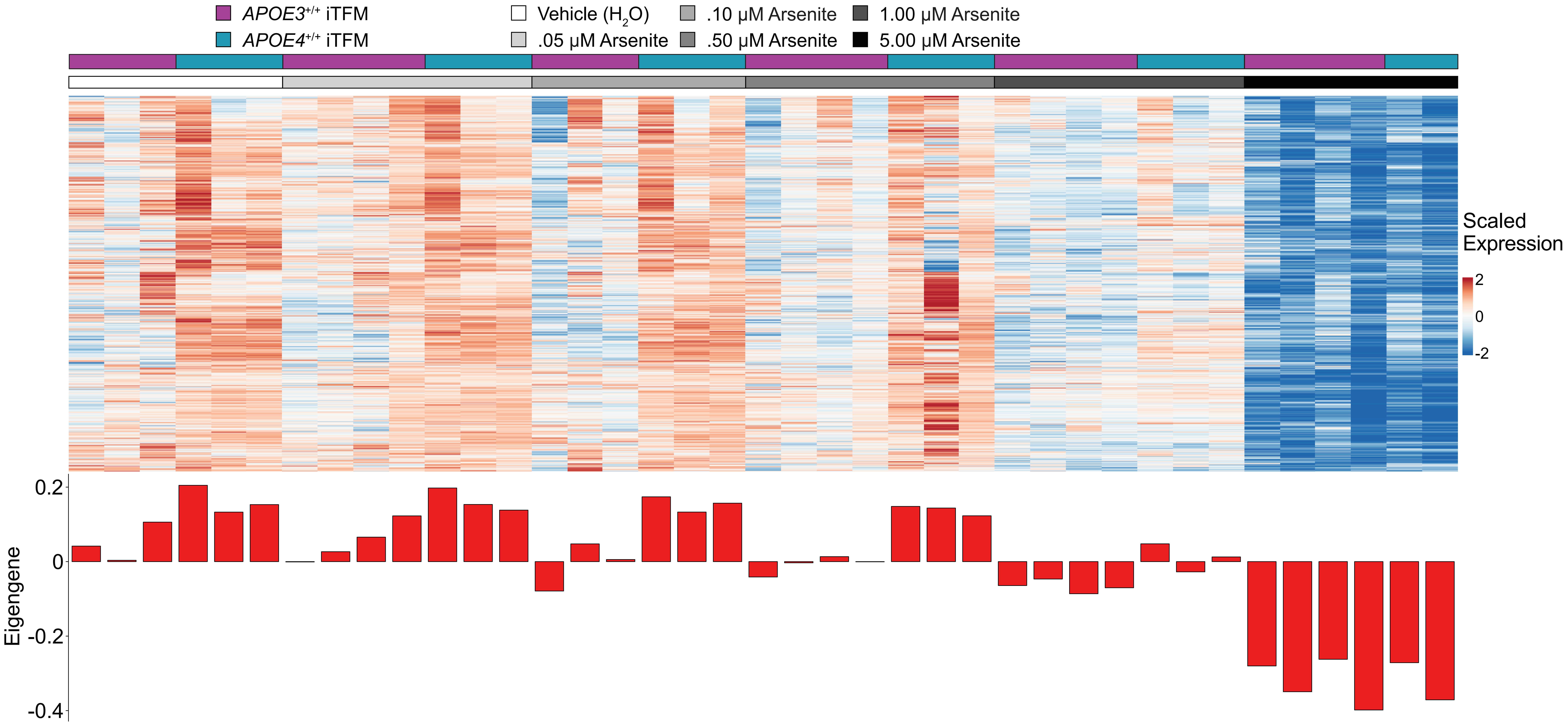
APOE3+/+ iTFM
APOE4+/+ iTFM
Vehicle (H_2O)
.05 μM Arsenite
.10 μM Arsenite
.50 μM Arsenite
1.00 μM Arsenite
5.00 μM Arsenite
Scaled Expression
2
0
-2
Eigengene
0.2
0
-0.2
-0.4

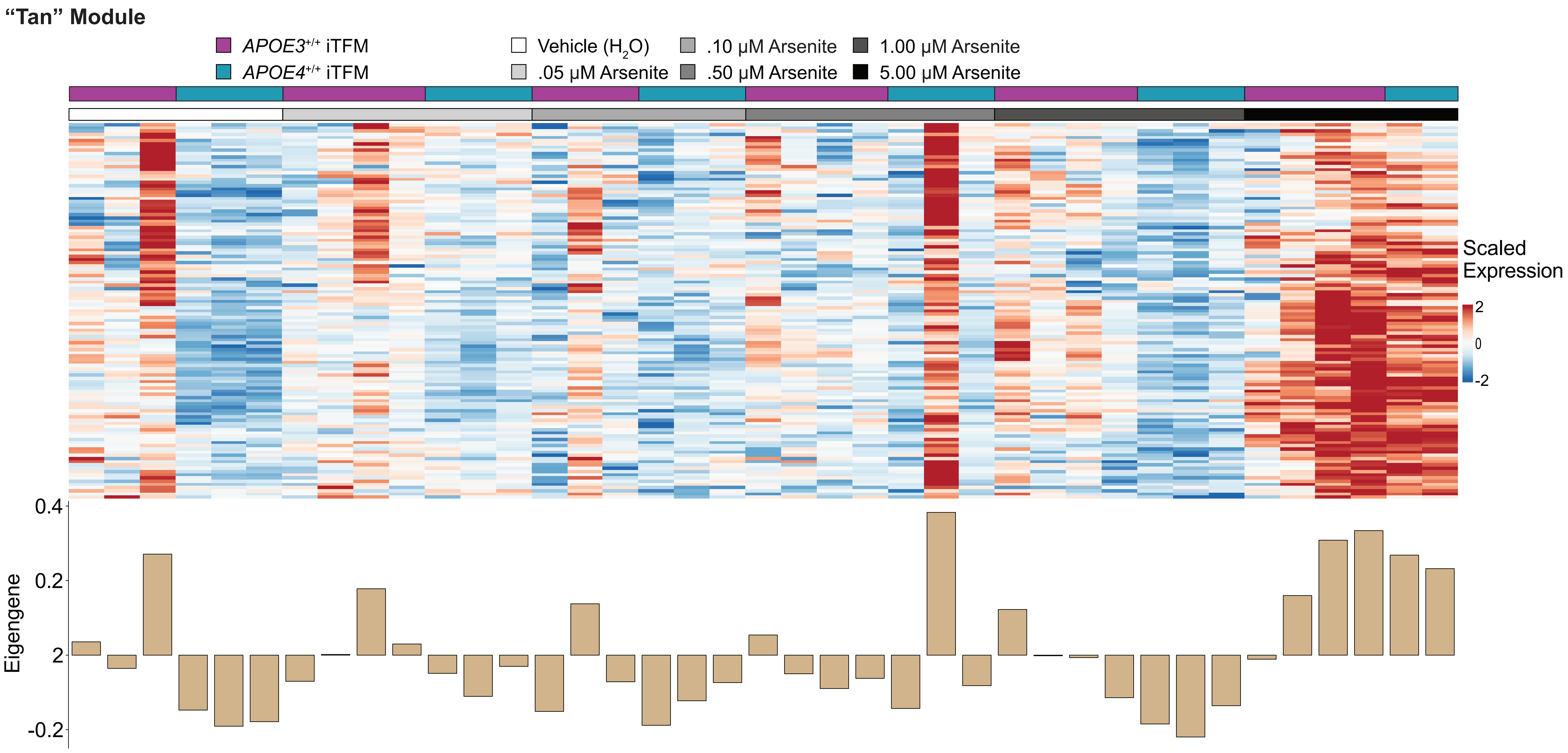

“Tan” Module
APOE3+/+ iTFM
APOE4+/+ iTFM
Vehicle (H2O)
.05 μM Arsenite
.10 μM Arsenite
.50 μM Arsenite
1.00 μM Arsenite
5.00 μM Arsenite
Scaled Expression
2
0
-2
Eigengene
0.4
0.2
2
-0.2

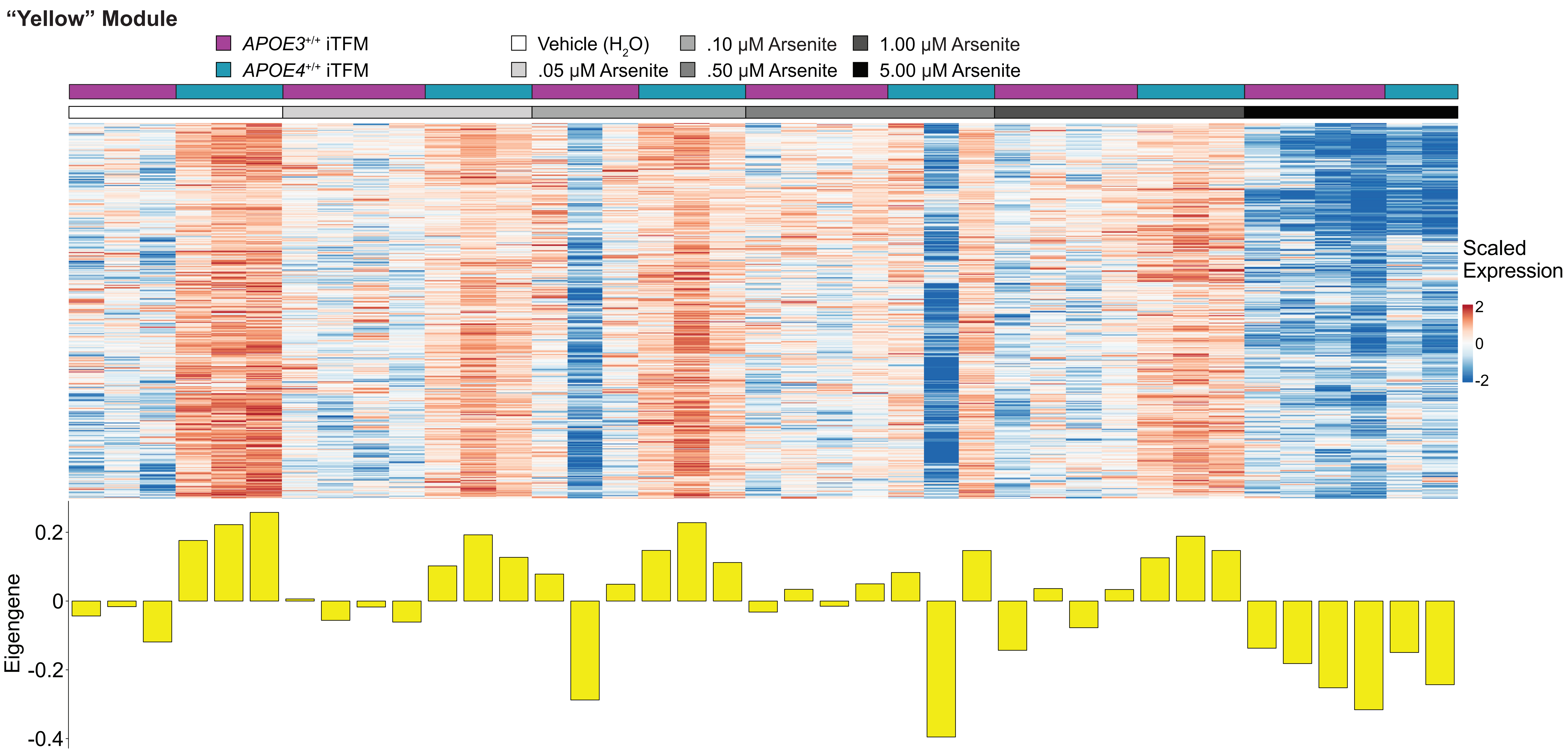
"Yellow" Module
APOE3+/+ iTFM
APOE4+/+ iTFM
Vehicle (H2O)
.05 μM Arsenite
.10 μM Arsenite
.50 μM Arsenite
1.00 μM Arsenite
5.00 μM Arsenite
Scaled Expression
2
0
-2
Eigengene
0.2
0
-0.2
-0.4
